# ADHD in children and adolescents: Review of current practice of non-pharmacological and behavioural management

**DOI:** 10.3934/publichealth.2023004

**Published:** 2023-02-07

**Authors:** Michael O Ogundele, Hani F Ayyash

**Affiliations:** 1 Department of Community Paediatrics, Bridgewater Community Healthcare NHS Foundation Trust, Halton District, UK; 2 Department of Integrated Paediatrics, Mid and South Essex University Hospitals Group, University Hospital NHS Foundation Trust, Southend-on-Sea, UK

**Keywords:** ADHD, Non-Pharmacological, behavioural, children, adolescents, evidence, psychoeducation, psychological interventions

## Abstract

Attention deficit Hyperactivity Disorder (ADHD) is the commonest childhood neurodevelopmental disorder, affecting 3 to 9% by school age, and often persists into adulthood. ADHD in children and young people (CYP) has wide ranging multi-modal impacts on the affected CYP, their carers and the society. Co-morbidity with other neurodevelopmental, behavioural and emotional disorders is the rule rather than exception. Pharmacological treatment is not recommended as the sole therapeutic intervention, and several other non-pharmacological interventions have been advocated within a framework of Multi-modal strategy as the norm, to address both the core symptoms as well as the behavioural and other related difficulties. All paediatric professionals need to be familiar with the principles of different modalities of non-pharmacological or behavioural interventions for managing ADHD in CYP. Most published up-to-date evidence for behavioural interventions both for the core ADHD symptoms and other outcome measures are summarized in this article, including the peculiar problems related to their research. The most effective evidence-based strategies for controlling ADHD core symptoms are combination of stimulant medications with Behavioural therapy (BT) or Cognitive behaviour therapy (CBT), as well as group-based parental Psychoeducation. Standalone BT, CBT, Mindfulness, Neurocognitive training and Neurofeedback cannot currently be recommended for controlling core symptoms due to limited evidence. Other Behavioural interventions could lead to improvements in ADHD-related outcomes, including parenting skills, CYP's social skills, academic performance and disruptive behaviours. School-based non-pharmacological interventions have been shown to reduce disruptive behaviours. Executive skills are also significantly improved with use of computer-based Neurocognitive training and regular physical Cardio exercises. It is disappointing that combinations of different types of psychosocial interventions have low efficacy on both the core ADHD symptoms and other related outcomes. The readers are welcome to test their knowledge and learning efficacy through an accompanying quiz.

## 1. Introduction

Attention-deficit/hyperactivity disorder (ADHD) is the commonest childhood neurodevelopmental disorder, affecting 3 to 9% by school age, and often persists into adulthood. Its core symptoms are persistent and pervasive inattention and/or overactivity/impulsiveness impairing functioning in multiple settings. It is now classified under the umbrella of “Neurodevelopmental disorders” within the DSM-5 and ICD-11 standards. Furthermore, 65–80% of patients with ADHD have conduct problems and other comorbidities, in addition to low academic achievement and poor social and organizational skills. Parents of children and young people (CYP) with ADHD often experience poor self-concept and emotional problems [1]. ADHD is also associated with significant psychosocial burden to the CYP, the family and wider society, including high cost of criminal justice involvement. It is therefore important to consider not just how to treat the core symptoms (for which medication is the mainstay) but also the behaviour problems which can result, as the latter lead to school exclusions and potentially academic failure [2].

Pharmacological treatment is not recommended as the sole therapeutic intervention, and several other non-pharmacological interventions have been advocated within a framework of Multi-modal strategy as the norm. In the UK, the National Institute for Clinical Excellence - NICE guidance recommends only psychosocial treatment for ADHD CYP with mild or moderate levels of symptoms and impairment, escalating to additional pharmacological treatment for those with severe symptoms and impairment, for those refusing non-pharmacological interventions or if the response is poor [2],[3]. Many carers and CYP with ADHD have reservations about use of medications for various personal and social reasons, including stigma. Studies based on prescription databases across several nations have shown that ADHD medication usage is significantly below the expected ADHD prevalence worldwide, apart from the USA and Iceland [2]. All clinicians therefore need to be familiar with the wide range of different non-pharmacological or psychological interventions for managing ADHD in children and young people (CYP), especially for families who are reluctant or sceptical about accepting pharmacological therapies.

We have assessed and summarise here the non-pharmacological interventions for ADHD which could be used as first line treatment for patients with ADHD. The implementation of non-pharmacological strategies has become particularly crucial during the COVID-19 pandemic crisis and thereafter, with restricted contact with healthcare professionals [4]. This article also explores the principles and published evidence of different non-pharmacological psychosocial interventions, providing a summary of most up-to-date evidence for their effectiveness. The literature search strategy is described in box A. We are discussing the effects of the non-pharmacological strategies on both the ADHD core symptoms and the associated behaviours separately, as they are two different important issues.

The readers are welcome to test their knowledge and learning efficacy through the accompanying quiz.

Clinical Scenario: A 14 year old boy was recently diagnosed with ADHD. He is reluctant to use stimulant medications because he and his parents have reservations about their side-effects. He has come to discuss with the Clinician about the effectiveness of non-pharmacological options for managing his symptoms.

## 2. Methods

We conducted a systematic search of the following electronic databases: Pubmed, PMC, CINAHL, Embase, PsycINFO, Ovid, Database of Abstracts and Review and the Cochrane Database of Systematic reviews, with no limitations in terms of language and date of publication from inception to Dec 2021. Search terms included a combination of the following: “non-pharmacolog?”, “childhood”, “psychoeducation”, “behavior?”, “parent?”, “ADHD”, “impairment” or “psycholog?”. The relevant publications were summarised, and the emerging themes are presented. Over 5000 abstracts and titles were retrieved from various databases searched, out of which relevant full text articles were independently identified by each author and included in the review by agreed consensus.

## 3. Results

### 3.1. Reasons for consideration of non-Pharmacological treatment

- Multimodal treatment approaches are recommended by several national and international clinical guidelines [3].
- Problems of public scepticism/cynicism about ADHD management
  Public opinions have become divided, and several controversies have emerged around the wide variations in medical and non-orthodox practices that have been promoted and implemented in the management of ADHD. Some people have gone as far as querying the existence of ADHD as a bona fide medical diagnosis [5].
- ADHD medications do not result in universal effective responses.
  It has been reported that 10–30% of CYP would not respond to ADHD stimulant medications [6]. Another 10% of ADHD children would be unable to tolerate the medications due to significant side effects [7]. Non-stimulant medications provide alternative options for pharmacological therapy, but they are generally less effective and not as widely studied compared to the stimulants [2],[8].
  Efficacy of stimulant medications is lower in younger children and is associated with a higher burden of side effects [9],[10]. Additionally, although core symptoms may respond, other important variables like academic achievement and social relationships can remain unchanged [2].
- Parental and some Clinicians' perceptions
  Some parents and clinicians are reluctant to recommend ADHD medication use due to concerns about undesirable long-term side-effects and express preference for non-medication options [11]. Some authors have reported 58% of carers refusing ADHD medications for their CYP with ADHD [12].
- Problematic low therapeutic compliance during adolescence.
- Pharmacotherapy is less effective for the management of commonest co-morbidities of oppositional and conduct problems or challenging behaviour.
- Pharmacologic treatment is less effective among preschool children [13].
- Adverse effects of pharmacological treatments
  Mild to moderate adverse effects are common with all pharmacological agents used in the treatment of ADHD, including disturbances of sleep, growth, appetite, blood pressure and heart rate. More severe side effects such as psychotic symptoms are also rarely reported. A few observational studies have also reported increased risks of seizures, tics, depression and suicidal attempts associated with pharmacologic treatment of ADHD [2].
- Higher level of acceptability
  Some studies have confirmed high levels of acceptability Behaviour therapy alone when compared to pharmacology treatment, and there are higher risks of patients discontinuing ADHD medication use compared to placebo [5].

### 3.2. Principles of non-Pharmacological treatments

Four types of Behavioural or Psychosocial interventions are available (Box 1). Each modality often involves implementation in several sessions over a lengthy period, through adult- (parents or teachers) or child-led interventions [14]. A central principle of non-pharmacological treatments is that measurable improvement in targeted neurocognitive functions could be achieved through intensive repetitive practice of prescribed procedures. The commonest non-pharmacological strategies are Psychoeducation, Neurocognitive training, Mindfulness-Based Interventions, Parent training and Behaviour therapy. These are defined and explained further in Box 2. Other complementary modalities such as Dietary therapy with nutritional supplements and Homeopathic treatment are excluded from this review due to limited evidence of efficacy.

Box 1. **Classification of modalities of non-Pharmacological/Psychological treatment of ADHD.**

1. Psychosocial interventions: (Behavioural therapy, parent education, peer-relationship-,and social skills-training and school/classroom-based interventions)
2. Body-focused interventions: (Body-oriented/yoga-based/physical exercise/sleep intervention/mindfulness-based interventions [MBI])
3. Cognitive/neuro-Cognitive Therapy (CT): CT/computer attention training/working-memory training/attention training/Neurofeedback (NF) training/Electromyographic (EMG) biofeedback interventions
4. Cognitive-behavioural training: (Psychoeducation, Play-therapy and Cognitive-Behaviour therapy)

Box 2. **Definitions of non-Pharmacological treatments.**

- Psychoeducation is defined as an intervention with systematic, structured and didactic knowledge transfer for an illness and its treatment, integrating emotional and motivational aspects to enable patients to cope with the illness and to improve its treatment adherence and efficacy. Psychoeducation empowers patients and families to better understand and cope with the illness more successfully and to commit to more long-term involvement.
- Neurocognitive training involves repeated practice of ADHD-specific deficient neuropsychological processes such as working memory or attention. Neurocognitive training typically involves digital, automated training exercises for strengthening any deficient neurocognitive function.
- Neurofeedback uses video visualization or sound representations of brain activity via EEG recording to teach children to increase attention and impulse control. The most frequently used frequencies in *Neurofeedback* enhance beta (15–18 Hz) and inhibit theta (4–7 Hz) brain activity.
- Mindfulness-Based Interventions enhance self-regulation and capacity to pay attention to experiences in the present moment through three processes: enhanced attention control, improved emotional regulation and altered self-awareness (meditation).
- Parent training encourages behaviour control strategies designed to enhance desirable and discourage inappropriate child behaviours, improve positive and constructive adult–child interactions, enhance Parenting self-concept and increase parenting confidence.
- Behavioural therapy includes interventions aimed at changing child's behaviours (enhancing appropriate behaviours and decreasing undesired behaviours), based on social learning and other cognitive principles.
- Cognitive Behaviour Therapy (CBT) integrates a combination of both cognitive and behavioural learning principles to teach coping skills and encourage desirable behaviour, emotions and thought patterns.

### 3.3. Problems with research into non-Pharmacological and psychological treatment effectiveness

Several problems have been identified as barriers to efforts in carrying out high quality research into the effectiveness of non-pharmacological and psychological treatment modalities, which are not encountered in research with pharmacological agents. These problems include difficulties with running truly blinded trials, high costs, a dearth of specialist therapists and homogeneity of ADHD phenotypes [11],[15],[16]. These are summarised in Box 3.

Box 3. **Problems with collating Evidence for effectiveness and limitations of non-Pharmacological treatment.**

- It is difficult to ensure the principles of gold-standard randomized trials with blinded outcome assessment (e.g., parents receiving parent training cannot be blinded), with the consequent bias risk of inflated efficacy estimates.
- Behavioural interventions produce outcomes that are difficult to maintain over long durations, and research projects are generally more expensive to run than pharmacological studies [15].
- The outcomes of behavioural interventions only affect specific targeted functions, do not appear to be generalised to other behaviours and may not be applicable in other settings [11].
- There is a general shortage of trained community-based behavioural specialists to match the number of CYP with ADHD.
- Significant improvement in some key areas of desirable functioning such as academic achievement, social skills, family relationships and executive abilities may not be achievable thorough non-pharmacological therapies [11].
- There are limited studies to confirm the sustenance of immediate treatment outcomes for many behavioural therapies (e.g., classroom behavioural management) [11].
- ADHD is an heterogeneous condition with impaired functioning in several neurocognitive domains, which are difficult to measure accurately.

### 3.4. Recent Evidence for effectiveness of non-Pharmacological treatment modalities

I. Treatment of ADHD core symptoms

The research evidence for non-pharmacological / psychological treatment for ADHD core symptoms is summarised in Table 1 and Box 4. Highest treatment effects for controlling ADHD core symptoms are reported for combination of stimulant medications with Behavioural therapy (BT) or Cognitive behaviour therapy (CBT) [15],[17]. There is also evidence for effectiveness of group-based parental Psychoeducation in improving ADHD core symptoms [18]. Other modalities such as Neurocognitive training (NCT) and Neurofeedback (NF) have shown less consistent effectiveness. A recent meta-analysis of 7 RCT of meditation-based therapies in CYP with ADHD showed high level of bias and poor quality of the studies. Low to moderate efficacy of the meditation-based interventions were reported for the core ADHD symptoms (Hedge's g = −0.44) compared to control conditions, but there was no significant effect on the neuropsychological measures of inattention and inhibition [19].

Box 4. **Summary of effectiveness of non-Pharmacological treatment modalities on treatment of ADHD core symptoms**

- Psychoeducation in group of parents improved ADHD symptoms and cognitive levels significantly [18].
- Behavioural therapy combined with stimulants seems superior to psychostimulants or non-stimulants alone.
- Stimulants alone seem superior to behavioural therapy, cognitive training and non-stimulants.
- Positive effects of Behavioural therapy are more likely to be reported for assessments made by unblinded parent ratings.
- Effectiveness of Cognitive behaviour therapy (CBT) for treating ADHD symptoms and functional impairment is mixed in several studies.
- Cognitive Behaviour Therapy (CBT) combined with stimulants improves core ADHD symptoms [17].
- The evidence for the effect of working memory training programmes in producing a significant decrease in ADHD symptomatology is mixed.
- Effectiveness of Neurocognitive training to improve ADHD symptoms and related functional impairments has been mixed in several studies.
- Cognitive training had significant effects on total ADHD and inattentive symptoms for reports by raters most proximal to the treatment setting (i.e., typically unblinded), while the effect size of cognitive training decreased to near 0 and became non-significant for blinded measurements made by observers.
- Meta-analytic evidence on the efficacy of Neurofeedback for ADHD core symptoms is currently mixed.

**Table 1.** Summary of evidence for treatment of ADHD core symptoms.

	**Single modalities**		**Combinations**

**Type of treatment**	**Effective outcomes**	**Mixed/Non-effective outcomes**	**Effectiveness**
**Behavioural therapy (BT)**	Moderate ES for typically unblinded parent ratings [20].	Effect size near 0 and non-significant for probably blinded measurements [20].	BT and stimulants: superior to stimulants or non-stimulants [5].
**Computer-based Cognitive training (CT)**	CT game for attention: reduction in the clinician ADHD-RS and functional EEG changes [31]. All types of CT: Significant effects on total ADHD ([SMD] = 0.37) and inattentive symptoms (SMD = 0.47) for unblinded raters [33].	CT for working memory: either no effect or mixed effects on ADHD symptoms [15],[32]. All types of CT: Small ES for total ADHD symptoms ([SMD] = 0.2) and inattentive symptoms (SMD = 0.32) for blinded raters [33]. All types of CT: No significant effects on H/I symptoms [33].	Stimulants and combined treatment groups with CT: More effective in improving ADHD symptoms [32].
**Cognitive Behaviour Therapy (CBT)**		Group-based CBT: Mixed effects on ADHD symptoms and functional impairment [34].	CBT and stimulants: improve core ADHD symptoms [17].
**NeuroFeedback [NF]**	Medium to large ES on inattention [SMD = 0.64 to 0.80), while ES for H/I was medium [SMD = 0.50–0.61) [35].	Effect is mixed for core ADHD symptoms, academic & social skills [36]–[38].	
**Psychoeducation**	Parents group Psychoeducation: Significant reduction of ADHD symptoms (p = 0.001) [18].		
**Meditation**	Low to moderate efficacy (Hedge's g = −0.44, 95% CI −0.69 to −0.19, I20%) compared to control conditions [19].		

*Note: BT – Behavioural therapy; CT – Computer-based Training; ES – Effect size (measured by SMD); H/I – Hyperactivity/Impulsivity symptoms; SMD – Standardized Mean Difference.

The reported effectiveness of standalone non-pharmacological or psychological modalities of BT, CBT, working memory training, Mindfulness (meditation), NGT and NF for controlling ADHD core symptoms is mixed, and they currently cannot be recommended as stand-alone therapies.

II. Effects on other ADHD-related behavioural problems.

**Table 2.** Summary of Evidence for other ADHD-related behavioural problems.

**Outcome measures**	**Single modalities**		**Combinations**

	**Effective treatments**	**Mixed/Non-effective treatments**	**Effectiveness**
**Parenting quality**	BT: Moderate ES for positive parenting (SMD = 0.68), negative parenting (SMD = 0.57) and small ES for parenting self-concept (SMD = 0.37) [20].		
**Parent mental health**		BT: no significant effects on self-rated mental health or general well-being [30].	
**Child conduct / functionality**	BT: Improved parental knowledge and children's emotional, social and academic functioning, reduced levels of oppositional and non-compliant behaviours [30],[39].	BT: Low ES on Child conduct problems (SMD 0.26), social skills (SMD 0.47) and academic performance (SMD 0.28) [20].	CBT with stimulants: Improve problematic and antisocial behaviours [17].
	Classroom interventions: reduced off-task and disruptive classroom behaviour [40].		
**Higher cognitive, e.g., Inhibition / Executive Functions (EF)**	Cardio exercise: improves EF-based events-related brain potentials; attention and academic performance [23],[41].		
	CT: improves EF compared to control children [22].		
	PSE: improved cognitive levels significantly [18].		
**Working memory (WM)**	Cogmed WM training: Improved verbal and visuo-spatial WM [32].		
**Weight loss in obesity**			Stimulants and EF training significantly improve the outcome of obesity [24].

*Note: BT – Behaviour Therapy; CBT – Cognitive Behaviour Therapy; CT – Computer-based Cognitive Training; EF – Executive Functioning; ES – Effect size; PSE – Psychoeducation; SMD – Standardized Mean Difference; WM – Working memory.

The research evidence for non-pharmacological /psychological treatment in controlling other ADHD-related behavioural problems is summarised in Table 2 and Box 5. Combination of stimulants with CBT improves problematic and antisocial behaviours [17].

Box 5. **Summary of effectiveness of non-Pharmacological treatment modalities on control of other ADHD-related behavioural problems.**

- Combination of stimulants with **Cognitive Behaviour Therapy (CBT**) improves problematic and antisocial behaviours [17].
- **Computer-based attention training game systems** significantly improve attention scores, and evidence of F-MRI captured Functionality of both the salience/ventral attention network (SVN) and dorsal attention network (DAN).
- **Cognitive training** improves Executive functions (EF), matching with the healthy control children [22].
- **Behavioural therapy** leads to significant improvements in 3 areas of **parenting quality:** positive parenting, decreased negative parenting and increased parenting self-concept.
- **Parent training and other Behavioural therapies** have shown positive results for parental knowledge and children's emotional, social and academic functioning - mostly from unblinded studies.
- **Parent training** improves effective parenting skills, reduces oppositional and noncompliant behaviours and may improve other functioning areas.
- **Parent training** has no significant treatment effects on self-rated **parental mental health** (depression/anxiety, general well-being).
- **Behavioural interventions** are effective with low effect sizes on Child Psychopathology and functionality including conduct problems, social skills and academic performance [20].
- Meta-analysis of 100 studies showed that classroom interventions reduce off-task and disruptive classroom behaviour in children with symptoms of ADHD, with largest effects for consequence-based and self-regulation interventions [16],[27].
- **Cardio exercise** improves executive functions based on event-related brain potentials; attention and Academic performance [23].

Parent-led and other adult/child-led Psychosocial interventions are associated with relatively low effect sizes, but they significantly improve Parenting skills, including positive parenting self-concept and improved parental knowledge [20]. They also lead to reduced levels of oppositional and non-compliant child behaviours, improving social skills and academic performances, but they do not significantly affect self-rated parental mental health (depression/anxiety, general well-being). Box 6 provides a list of evidence-based non-pharmacological resources for parents of CYP with ADHD [21].

Box 6. **Examples of online resource materials for non-Pharmacological parent-based Behavioural interventions.**

**Table:** 

**Behaviour Programme**	**Age Range**	**Website**
Positive parenting program (triple P)	Birth 0–12 years; teen triple P: 12–16 years	www.triplep.net
Parent child interaction therapy (PCIT)	2–8 years	www.pcit.org
Incredible Years	Parent based: 0–12 years; teacher based: 3–8 years	www.incredibleyears.com
Kazdin method (Parent Management Training – PMT)	6–12 years	www.alankazdin.com / https://www.parentmanagementtraininginstitute.com/
New Forest parenting programme	3–13 years	www.guidebook.eif.org.uk
Defiant Teens	13–18 years	https://www.guilford.com/books/Your-Defiant-Teen/Barkley-Robin/9781462511662
Problem-solving skills training (PSST)	6–14 years	https://www.parentmanagementtraininginstitute.com/treatment-programs1.html
Behavioural and Emotional Skills Training (BEST)	All Ages	https://childmind.org/center/behavioral-and-emotional-skills-training/
Tuning in to Kids	4–6 years	https://tuningintokids.org.au/
Family Check-up for Children	2–17 years	https://guidebook.eif.org.uk/programme/family-check-up-for-children
Common Sense Parenting	6–16 years	https://youth.gov/content/common-sense-parenting
Centres for Disease Control and Prevention (CDC) Information for Parents	All Ages	https://www.cdc.gov/ncbddd/adhd/behavior-therapy.html
National Institute for Health and Care Excellence (NICE)	Psychosocial interventions for antisocial behaviours	www.nice.org.uk/guidance/cg158
HelpGuide Parenting tips	All ages	https://www.helpguide.org/articles/add-adhd/when-your-child-has-attention-deficit-disorder-adhd.htm

Classroom-based behavioural and teacher-based psychoeducation interventions are reported to reduce disruptive classroom behaviour in CYP with ADHD.

Executive functioning in CYP with ADHD including attention and Academic performance are significantly improved with use of digital attention training game systems and other NCT interventions and active involvement in regular Cardio exercises [8],[22],[23]. Limited studies are showing preliminary results that provide evidence for improved weight loss in CYP with ADHD and comorbid obesity with combined pharmacotherapy and executive function training [24].

III. Study of combined non-pharmacological modalities.

Research studies on combined modalities, e.g., Computer-based Cognitive Training (CT) and parent training and combined CT, physical exercises and classroom behaviour management strategies, have yielded disappointingly no significant improvements on either the ADHD core symptoms or other related outcomes [25],[26].

## 4. Discussion

There is an accumulated body of published evidence to prove the efficacy of some behavioural / psychosocial strategies for managing CYP with ADHD, with measurable benefits on several domains of daily patient and parent functioning. The only non-pharmacological intervention that has consistently shown significant benefits in several studies is parent- or teacher-child dyad Behavioural therapy [5]. Significant beneficial effects of classroom-based Behaviour-modification strategies for managing ADHD-related behavioural and academic problems have been well documented [16],[27].

Several ADHD-related outcomes, including parenting skills, child's social skills, academic performances and challenging behaviours, have been shown to respond significantly to non-pharmacological, psychological and Behavioural treatment strategies. School-based non-pharmacological interventions have been shown to reduce disruptive behaviours. Executive skills are also significantly improved with use of computer-based Neurocognitive training and regular physical Cardio exercises [22],[23].

It is important to acknowledge that most clinical studies of ADHD in CYP are based on a predominantly male population, which has been partly attributed to gender stereotyping and symptomatology differences [28]. The effectiveness of various non-pharmacological therapies may therefore need to be interpreted with caution among young females with ADHD, who are more likely to present with psychological comorbidities [29]. The list of non-pharmacological therapies reviewed in this paper are listed in Table 3 along with a summary of their effectiveness and the current recommendations for their clinical implementation.

**Table 3.** Summary of clinical recommendations for the non-pharmacological methods.

**Type of treatment**	**Effect on ADHD Core symptoms**	**Effect on Related Behaviour problems**	**Clinically Recommended Alone**
**Combined Stimulants and Behavioural therapy (BT)**	Superior to medications only [5].	Moderate to High ES	**YES**
**Combined Stimulants and Cognitive training (CT)**	More effective in improving ADHD symptoms [32].	Moderate to High ES	**YES**
**Combined Stimulants and Cognitive Behaviour Therapy (CBT)**	More effective in improving ADHD symptoms [17].	Improve problematic and antisocial behaviours [17].	**YES**
**Stimulants and Executive Function (EF) training**	Superior to medications only	Significantly improve obesity management [24].	**YES**
**Behavioural therapy (BT)**	Zero to Moderate ES [20].	No significant effects on self-rated mental health or general well-being [30]. Low to Moderate effects on parental knowledge, parenting style, children's emotional, social and academic functioning, reduced levels of challenging behaviours [20],[30],[39].	**NO**
**Computer-based CT (All types)**	Zero to Significant effects on core symptoms [33].	Improves EF [22].	**NO**
**CT game for attention**	reduction in core symptoms [31].	reduction in functional EEG changes [31].	**NO**
**CT for working memory (WM)**	Either no effect or mixed effects on core symptoms. [15],[32]	Improved verbal and visuo-spatial WM [32].	**NO**
**Cognitive Behaviour Therapy (CBT)**	Zero to Moderate ES [34].	Zero to Moderate effects on functional impairment [34].	**NO**
**NeuroFeedback [NF]**	Zero to large ES on core symptoms [35].	Effect is mixed for academic & social skills [36],[37].	**NO**
**Parental Psychoeducation**	Significant reduction of core symptoms [18].	improved cognitive levels significantly [18].	**NO**
**Classroom interventions**		Reduced off-task and disruptive behaviours [40].	**NO**
**Cardio exercise**	Improve attention and academic performance [23],[41].	Improves EF-based events-related brain potentials [23],[41].	**NO**
**Meditation**	Low to moderate efficacy [19].		**NO**

*Note: BT – Behaviour Therapy; CBT – Cognitive Behaviour Therapy; CT – Cognitive Training; EF – Executive Functioning; ES – Effect size; WM – Working memory.

## 5. Evaluation of the review methodology

Standard procedures for evaluating quality of the published evidence were not applied in this brief paper because it was not designed to be a systematic review. This is partly due to the dearth of high-quality research studies available for many non-pharmacological therapies and interventions. This article is mainly addressed to busy clinicians making daily judgment about appropriate psychosocial interventions for their young ADHD patients and their carers.

## 6. Future research directions

With new advancements in computer science and wider implementation of Artificial Intelligence and Machine learning in many sectors of human endeavours, we could anticipate that more sophisticated neurocognitive enhancement programmes will be based on new advances in ADHD Neurobiology and consequently lead to better targeted improvements in both the core symptoms and other behavioural effects of ADHD [11].

We need to explore further ways to individualise optimal treatment for CYP with such a heterogenous condition as ADHD and in the future tackle potential public scepticism and cynicism regarding its management [30].

## 7. Conclusions

Combining behavioural therapy with stimulants has shown significant effectiveness in enhancing attentional processes, reduces impulsiveness and may potentially lead to reduced dosing and duration of medication treatment and also help to reduce the risk of side effects. It also improves problematic and antisocial behaviours [15],[17]. Studies of various combined modalities of psychosocial treatment have been disappointing, resulting in outcomes of poor dose-response effects [25],[26].

Standalone BT, CBT, Mindfulness, Neurocognitive training and Neurofeedback cannot currently be recommended (Table 3) for controlling core symptoms due to limited evidence [5].

Parenting CYP with ADHD can be very stressful, and targeting parental mental health problems with appropriate additional service provisions may enhance the effects of behavioural interventions [27]. More widespread availability of non-pharmacological interventions can lead to more widespread recommendation to CYP with ADHD through “social prescribing” by Clinicians (Box 6) and through various Local Authority “Offers” in the UK.

The limitation of this paper should be noted because it was not designed as a systematic review, with no strict evaluation of the quality of the published evidence. The recommendations of this article are designed as a quick reference for busy clinicians for making quick day-to-day decisions for managing CYP with ADHD and supporting their carers. The recommendations will continue to evolve in the future as more robust research evidence becomes available.
