# Supplementary material for: ADHD in children and adolescents: Review of current practice of non-pharmacological and behavioural management
Source: AIMS Public Health. 2023 Feb 7;10(1):35–51. doi: 10.3934/publichealth.2023004 (PMC10091126; doi:10.3934/publichealth.2023004)
Supplement: Supplementary file 1 [file publichealth-10-01-004-s001.pdf]

Public Health

AIMS Public Health, 10(1): 35–51.
DOI: 10.3934/publichealth.2023004
Received: 08 September 2022
Revised: 15 January 2022
Accepted: 28 January 2023
Published: 07 February 2023

http://www.aimspress.com/journal/aimsph

Review

ADHD in children and adolescents: Review of current practice of non-pharmacological and behavioural management

Michael O Ogundele[1,]* and Hani F Ayyash[2]

1 Department of Community Paediatrics, Bridgewater Community Healthcare NHS Foundation Trust, Halton District, UK
2 Department of Integrated Paediatrics, Mid and South Essex University Hospitals Group, University Hospital NHS Foundation Trust, Southend-on-Sea, UK

* **Correspondence**: Email: M.Ogundele@nhs.net.

Appendix:

Box 6 – QUIZ

(Quiz also available online: https://www.classmarker.com/online-test/start/?quiz=je95d6acda4ce44b OR https://tinyurl.com/y4lo6jha)

Questions
**1. What aspects of life are affected by ADHD?**<br>a) Academic difficulties<br>b) Social and communication skills<br>c) Parent-child relationships<br>d) Quality of life and emotional difficulties<br>e) Economic burden<br>f) All of the above
**2. What proportion of children with ADHD have one or more co-morbid conditions?**<br>a) 15% to 30%<br>b) At least 65%<br>c) More than 90%

d) None
**3. What are the impacts of ADHD on School performance?**<br>a) Lower risk of executive function deficit in ADHD<br>b) Increased risk of poor academic performances, for repeating grade and requiring extra help<br>c) Behavioural problems can lead to suspension and, ultimately, exclusion from school<br>d) Reduced risk of specific learning disorders such as dyslexia or dyscalculia<br>e) Increased likelihood of being involved in bullying behaviour at school
**4. Modalities of non-Pharmacological treatment include:**<br>a) Parenting training<br>b) Psychoeducation<br>c) Guanfacine<br>d) Neurofeedback<br>e) Classroom Behavioural strategies<br>f) Cognitive Behaviour Therapy
**5. Rationale for non-Pharmacological treatment include:**<br>a) 10–30% of ADHD patients show partial or no response to different medications<br>b) Up to 58% of parents refuse stimulant medication for their ADHD children and preference for alternative (non-medication) treatments<br>c) Stimulant medications are more effective and better tolerated by preschool children with ADHD<br>d) Stimulants may not improve important aspects of functional impairment (e.g., academic achievement).<br>e) Adverse effects on sleep, appetite, and growth and poor compliance can limit effectiveness and tolerance of medications.<br>f) Pharmacotherapy is less effective for the management of commonest co-morbidities of oppositional and conduct problems and challenging behaviour
**6. Aetio-pathogenesis of challenging behaviour in children arises from:**<br>a) Chronic stresses of living in poor socio-economic environments<br>b) Attending poor-performing schools<br>c) Familial heritable factors<br>d) Dysfunctional coercive parent-child interactional cycles<br>e) Non-nutritional diet
**7. What factors make non-Pharmacological / Psychological treatment less amenable to rigorous research and evaluation?**<br>a) Interventions are often more difficult to sustain over long durations are generally more costly<br>b) Outcome assessmentis less likely to be subject to blinding as in RCT<br>c) Specific interventions may not be generalized to non-targeted settings or domains<br>d) Inadequate availability of Therapists and Specialists<br>e) Studies are easier to conduct and evaluate<br>f) Heterogeneity in aetio-pathogenesis and manifestations of ADHD in children and adolescents

8. **Definition and explanation of non-Pharmacological treatments:**
   a) ***Neurofeedback*** uses video visualization or sound representations of brain activity via EEG recording to teach children to increase attention and impulse control.
   b) The most frequently used frequencies in ***Neurofeedback*** enhance beta (15-18 Hz) and inhibit theta (4-7 Hz) brain activity.
   c) **Psychoeducation** empowers patients and family to better understand and cope with the illness more successfully and to commit to more long-term involvement.
   d) **Neurocognitive training** involves repeated memorization of favourite rhymes and songs to enhance working memory or attention.
   e) **Mindfulness-Based Interventions** enhance self-regulation and capacity to pay attention to experiences in the present moment through three processes: attention control, emotional regulation, and altered self-awareness (meditation).

9. Principles of **Pharmacological / Psychological treatment:**
   a) ***Behavioural therapy*** aims to change behaviours, based on social learning principles and other cognitive theories.
   b) **Neurocognitive training** typically involves computer-based, automated training exercises designed to strengthen deficient neurocognitive functions.
   c) A central principle of **non-Pharmacological** treatments is that extensive practice, repetition, and feedback that results in lasting improvement in targeted neurocognitive functions.
   d) **Parent / Teacher training** encourages behaviour modification techniques to reinforce appropriate and discourage inappropriate child behaviours
   e) Child-focused ***Behavioural therapy*** interventions include organisational skills training
   f) **Non-Pharmacological** treatments are usually offered in single sessions either through training the adult parents/ teachers or the child or both.

10. Effects of Non-Pharmacological / Psychological treatment ADHD on core symptoms:
   a) **Behavioural therapy** in combination with stimulants is superior to stimulants or non-stimulants alone.
   b) Stimulants alone is inferior to **behavioural therapy, cognitive training** and non-stimulants
   c) Positive effects of **Behavioural therapy** are more likely to be reported for assessments made by individuals most proximal to the therapeutic setting—typically unblinded parent ratings.
   d) Effectiveness of **Cognitive behaviour therapy** for treating ADHD symptoms and functional impairment is mixed in several studies.
   e) **Neurocognitive training** has been shown to improve ADHD symptoms when outcomes were provided by in blinded raters.
   f) Meta-analytic evidence on the efficacy of **NeuroFeedback** for ADHD core symptoms is very strong.

11. **Effects of Non-Pharmacological / Psychological treatment on** other **ADHD-related behavioural problems:**
   a) **Computer-based attention training game system** significantly improve attention scores and evidence of f-MRI captured functionality.

b) **Behavioural therapy** leads to significant improvements in 3 areas of parenting quality**:** positive parenting, decreased negative parenting and increased parenting self-concept.
c) **Parent training** improves parenting, reduces levels of oppositional and noncompliant behaviours and may improve other aspects of functioning.
d) Meta-analysis of 100 studies showed that **classroom interventions** reduce off-task and disruptive classroom behaviour in children with symptoms of ADHD.
e) **Parent training and other Behavioural interventions** have shown positive results in relation to parental knowledge, children's emotional, social and academic functioning - although most studies have not used blinded outcomes.
f) **Parent training** have no significant effects of treatment on self-rated parent mental health (depression/anxiety, general well-being).

12. Which of the following statements are true?

a) There is evidence that behavioural interventions used to treat children and adolescents with ADHD had beneficial effects on important aspects of child and parent functioning.
b) Behavioural therapy, particularly given by parents and with active child and teacher involvement, is associated with statistically significant benefits.
c) Cognitive training, neurofeedback, dietary therapy (such as restricted elimination diet), polyunsaturated fatty acids, amino acids, minerals, and physical activity cannot be recommended as evidence-based interventions for ADHD symptoms until better evidence is available.
d) Combining behavioural therapy with stimulants may enhance attention, reduce impulsiveness, and may help reduce the dose and duration of stimulants.
e) UK NICE recommends non-pharmacological treatment for ADHD children with mild or moderate levels of symptoms and impairment.

Answers:
1. f (T);
2. a. (F), b. (T), c. (F), d. (F);
3. a. (F), b. (T), c. (T), d. (F), e. (T);
4. a. (T), b. (T), c. (F), d. (T), e. (T), f. (T);
5. a. (T), b. (T), c. (F), d. (T), e. (T), f. (T);
6. a. (T), b. (F), c. (T), d. (T), e. (F);
7. a. (T), b. (T), c. (T), d. (T), e. (F), f. (T);
8. a. (T), b. (T), c. (T), d. (F), e. (T);
9. a. (T), b. (T), c. (T), d. (T), e. (T), f. (F);
10. a. (T), b. (F), c. (T), d. (T), e. (F), f. (F);
11. a. (T), b. (T), c. (T), d. (T), e. (T), f. (T);
12. a. (T), b. (T), c. (T), d. (T), e. (T);

AIMS Press
© 2023 the Author(s), licensee AIMS Press. This is an open access article distributed under the terms of the Creative Commons Attribution License (http://creativecommons.org/licenses/by/4.0).
